# Supplementary material for: Optimized Fast Filtration-Based Sampling and Extraction Enables Precise and Absolute Quantification of the Escherichia coli Central Carbon Metabolome
Source: Metabolites. 2023 Jan 18;13(2):150. doi: 10.3390/metabo13020150 (PMC9965072; doi:10.3390/metabo13020150)
Supplement: Supplementary file 1 [file metabolites-13-00150-s001.zip › Supplementary Figure S1-Thorfinnsdottir et al.pdf]

Supplementary Figure S1: PES filters contaminate high-resolution mass spectra when submerged in organic solvents

Polyethersulfone (PES, 61110, Pall, Port Washington, NY, USA) and polyvinylidene fluoride (PVDF, HVLP04700, Sigma-Aldrich, Saint-Louis, MO, USA) membrane disk filters with a diameter of 47 mm were submerged in acetonitrile (ACN):water (1:1, *v*/*v*) and extracted and concentrated for downstream mass spectrometric analysis as described in [1]. Non-targeted analyses of the extracts were performed on a Waters ACQUITY I-Class UPLC coupled to a Waters Synapt G2-S Q-TOF high-definition mass spectrometer, applying HILIC separation with negative electrospray ionization (ESI-).

Lyophilized extracts were resuspended in water:ACN (1:9, *v*/*v*%) and injected (5 µl) onto an ACQUITY UPLC BEH Amide 2.1 * 100 mm column (186004801, Waters Millford, MA, USA) fitted with an ACQUITY BEH Amide VanGuard 2.1 * 5 mm pre-column (186004799, Waters) both with a pore size of 1.7 µm. The column was maintained at 40 °C and eluted with mobile phases (A) water:ACN (6:4, *v*/*v*%) and (B) water:ACN (10:90, *v*/*v*%), both added 10 mM ammonium hydroxide (5.33005.0050, Sigma Aldrich), pH 9. The following gradient was applied with a flow rate of 0,4 mL/min: 0-1 min: 99% B, 3-3,5 min: 99-78% B, 3,5-3,6 min: 78-99% B; 3,6-6,5 min: 99% B. The mass spectrometer was operated in MS^E mode, and the collision energy was ramped from 20 to 35 eV. The resolution of the mass spectrometer was 20 000, and data were acquired over the mass range of 50 – 1500 Da.

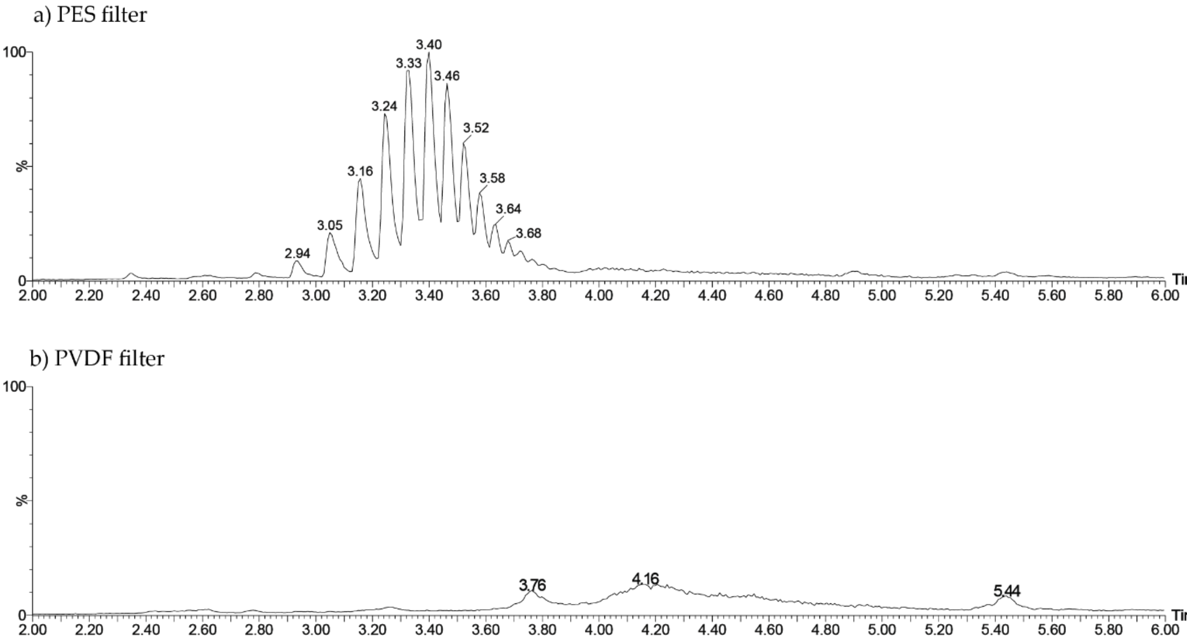

Supplementary Figure S1: HILIC ESI- Q-TOF MS^E total ion chromatograms from the analysis of acetonitrile (ACN):water extracts of **(a)** polyethersulfone (PES) and **(b)** polyvinylidene fluoride (PVDF) membrane disk filters. X-axes are scaled to the peaks with the highest intensity.

References

1. Røst, L.M.; Thorfinnsdottir, L.B.; Kumar, K.; Fuchino, K.; Langørgen, I.E.; Bartosova, Z.; Kristiansen, K.A.; Bruheim, P. Absolute Quantification of the Central Carbon Metabolome in Eight Commonly Applied Prokaryotic and Eukaryotic Model Systems. *Metabolites* **2020**, *10*, doi:10.3390/METABO10020074.
