# Supplementary material for: Optimized Fast Filtration-Based Sampling and Extraction Enables Precise and Absolute Quantification of the Escherichia coli Central Carbon Metabolome
Source: Metabolites. 2023 Jan 18;13(2):150. doi: 10.3390/metabo13020150 (PMC9965072; doi:10.3390/metabo13020150)
Supplement: Supplementary file 1 [file metabolites-13-00150-s001.zip › Supplementary Figure S2-Thorfinnsdottir et al.pdf]

Supplementary Figure S2: Chromatograms of *E. coli* extracts rinsed with water or an ionic rinsing solution

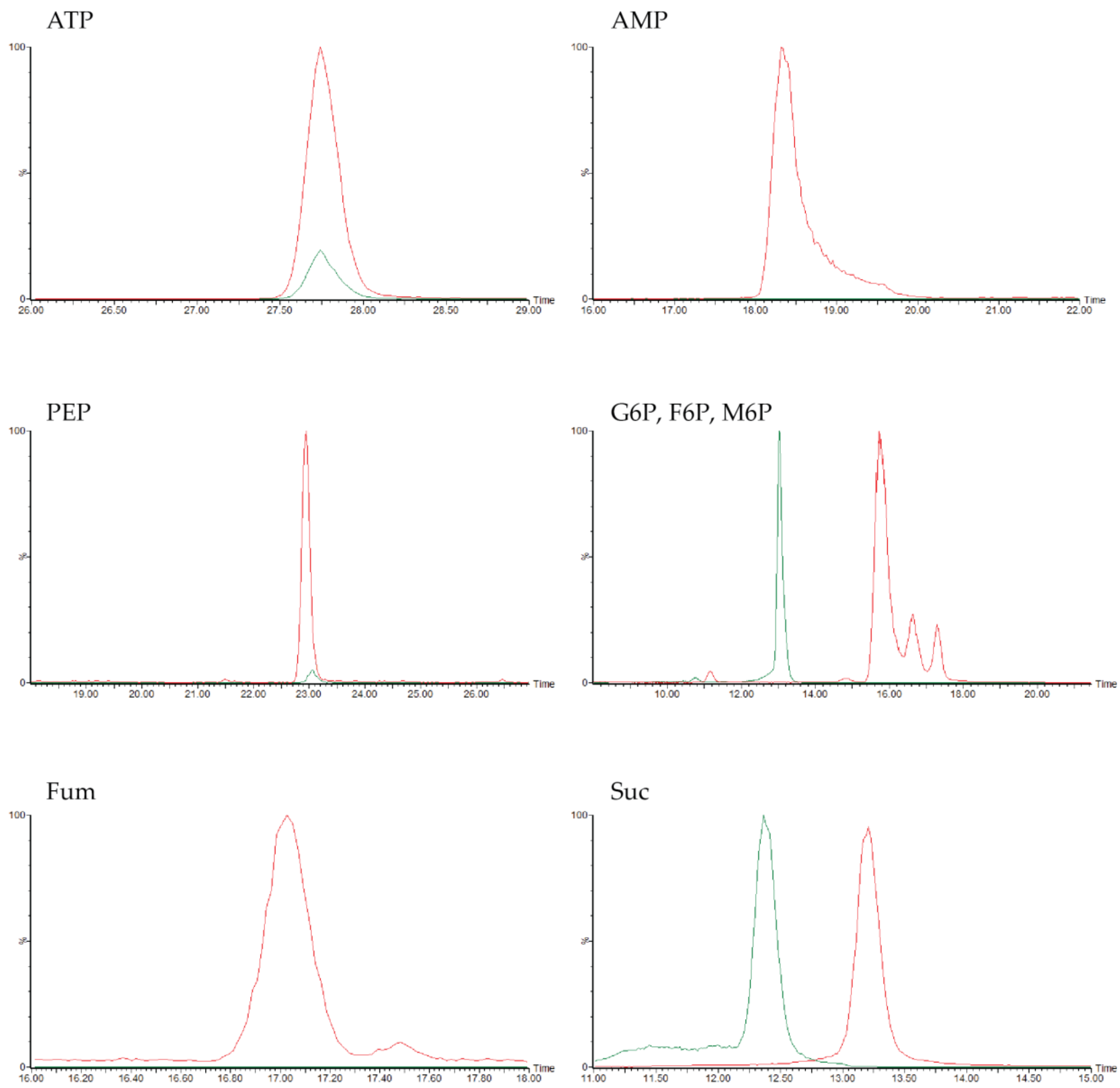

Supplementary Figure S2: Selected capillary ion chromatography tandem mass spectrometry (capIC-MS/MS)-chromatograms from the analysis of *E. coli* rinsed with different warm solvents. Chromatograms of extracts subjected to a warm (37 °C) ionic rinse (green) and warm (37 °C) water rinse (red) are superimposed, and the x-axes are scaled to the peaks with the highest intensity. The signal was < the limit of detection for AMP and fumarate subjected to the ionic rinse. PEP, phosphoenolpyruvic acid; G6P, glucose 6-phosphate; F6P, fructose 6-phosphate; M6P, mannose 6-phospate; Fum, fumarate; Suc, succinate.
