# Supplementary material for: Optimized Fast Filtration-Based Sampling and Extraction Enables Precise and Absolute Quantification of the Escherichia coli Central Carbon Metabolome
Source: Metabolites. 2023 Jan 18;13(2):150. doi: 10.3390/metabo13020150 (PMC9965072; doi:10.3390/metabo13020150)
Supplement: Supplementary file 1 [file metabolites-13-00150-s001.zip › Supplementary Figure S3-Thorfinnsdottir et al.pdf]

Supplementary Figure S3: Representative growth curves of *Escherichia coli* cultivated under three different conditions

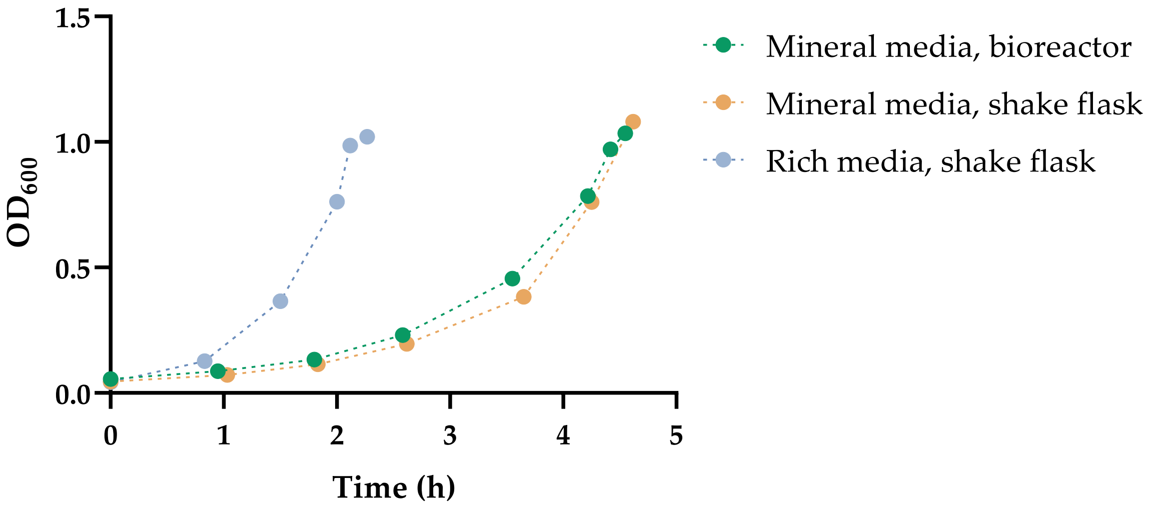

Supplementary Figure S3: Representative growth curves of *E. coli* cultivated under three different conditions; stirred benchtop bioreactors with mineral media, shake flasks with mineral media, and shake flasks with rich media.
