# Supplementary material for: Optimized Fast Filtration-Based Sampling and Extraction Enables Precise and Absolute Quantification of the Escherichia coli Central Carbon Metabolome
Source: Metabolites. 2023 Jan 18;13(2):150. doi: 10.3390/metabo13020150 (PMC9965072; doi:10.3390/metabo13020150)
Supplement: Supplementary file 1 [file metabolites-13-00150-s001.zip › Supplementary Figure S4 - Thorfinnsdottir et al.pdf]

**Supplementary Figure S4: Most trends are conserved across commonly applied normalization strategies for metabolite data**

*Escherichia coli* metabolite extract concentrations were normalized by two different strategies, either to experimental bacterial cell counts and bacterial cell volumes from flow cytometry and microscopy, respectively to obtain intracellular concentrations (mol/L), or to cell dry weight (CDW) measurements. Principal component analyses (PCA) were performed to investigate whether the choice of normalization strategy would influence interpretation of trends in the dataset, which it did not.

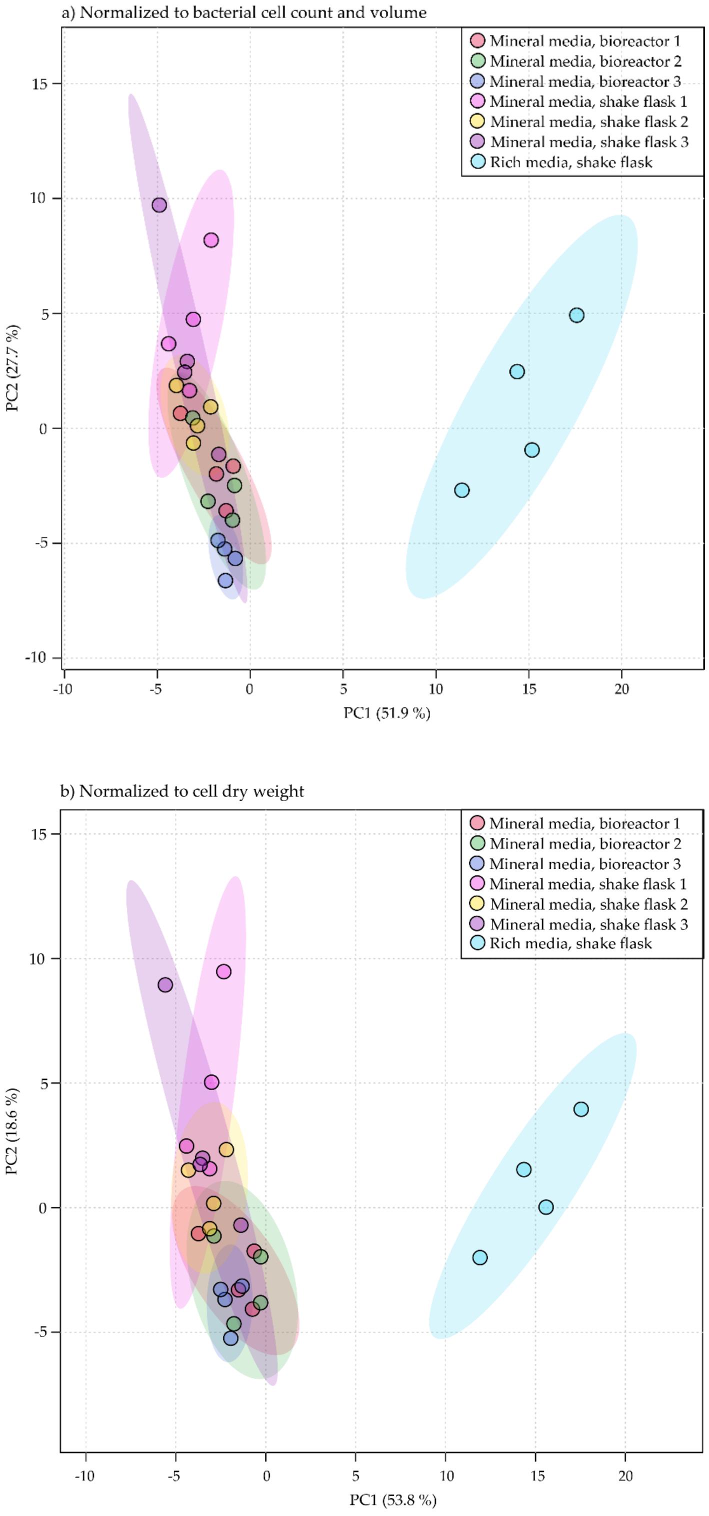


**Supplementary Figure S4:** Scores plot from principal component analyses (PCA) of n = 4 technical replicates from 1-3 biological replicates of *Escherichia coli* cultured in three commonly applied cultivation setups. Extract concentrations were normalized to **(a)** bacterial cell counts and bacterial cell volume to obtain intracellular concentrations (mol/L) or to **(b)** cell dry weight (CDW) to obtain mol/g CDW.
