# Supplementary material for: Optimized Fast Filtration-Based Sampling and Extraction Enables Precise and Absolute Quantification of the Escherichia coli Central Carbon Metabolome
Source: Metabolites. 2023 Jan 18;13(2):150. doi: 10.3390/metabo13020150 (PMC9965072; doi:10.3390/metabo13020150)
Supplement: Supplementary file 1 [file metabolites-13-00150-s001.zip › Supplementary Figure S5 - Thorfinnsdottir et al.pdf]

Supplementary Figure S5: Subtracting the background signal does not affect the distribution between metabolite classes

Metabolite extract concentrations in spent media samples from *Escherichia coli* were subtracted from metabolite extract concentrations in samples containing biomass collected at the same time point. The contributions (%) of different metabolite classes to the total quantified central carbon metabolite pool were calculated to verify that the distribution between metabolite classes was not affected by the media background signal.

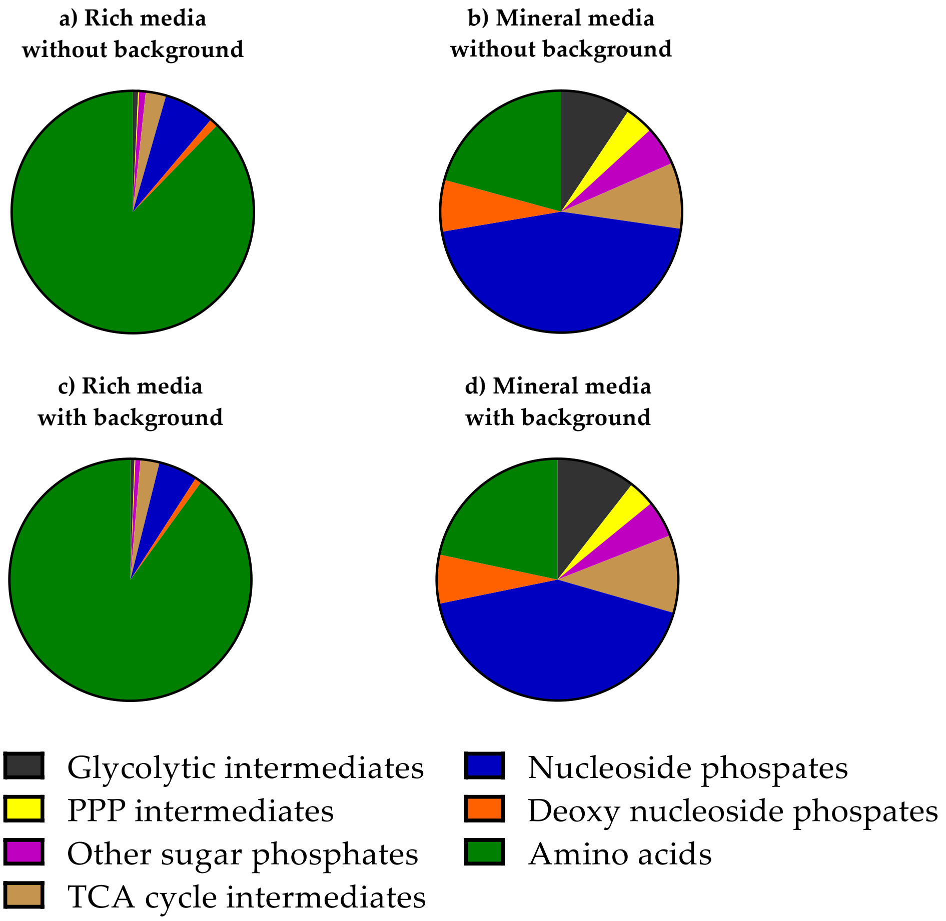

Supplementary Figure S5: Contributions (%) of different metabolite classes to the total quantified central carbon metabolite pool in *Escherichia coli* shake flask cultures in **(a)** rich or **(b)** mineral medium after subtraction of the background signal, and in **(c)** rich or **(d)** mineral medium without subtraction of the background signal. The averages of n = 4 technical replicas from one (a and c) or three (b and d) biological replicates are presented. TCA; tricarboxylic acid, PPP; pentose phosphate pathway.
