# Supplementary material for: Optimized Fast Filtration-Based Sampling and Extraction Enables Precise and Absolute Quantification of the Escherichia coli Central Carbon Metabolome
Source: Metabolites. 2023 Jan 18;13(2):150. doi: 10.3390/metabo13020150 (PMC9965072; doi:10.3390/metabo13020150)
Supplement: Supplementary file 1 [file metabolites-13-00150-s001.zip › Supplementary Table S1 - Thorfinnsdottir et al.pdf]

Table S1 Metabolite abbreviations: Metabolites included in panel for metabolite profiling, sorted on metabolite class and listed with abbreviation and CAS number. TCA; tricarboxylic acid, PPP; pentose phosphate pathway.

Class	Abbreviation	Metabolite	CAS number
Glycolysis	G6P	Glucose 6-phosphate	56-73-5
	F6P	Fructose 6-phosphate	643-13-0
	F1,6BP	Fructose 1,6-bisphosphate	488-69-7
	2PG	2-Phosphoglyceric acid	2553-59-5
	3PG	3-Phosphoglyceric acid	820-11-1
	PEP	Phosphoenolpyruvic acid	138-08-9
PPP	6PG	6-Phosphogluconic acid	921-62-0
	R5P	Ribose 5-phosphate	3615-55-2
	S7P	Sedoheptulose 7-phosphate	2646-35-7
Other sugar phosphates	F1P	Fructose 1-phosphate	15978-08-2
	G1P	Glucose 1-phosphate	59-56-3
	M1P	Mannose 1-phosphate	27251-84-9
	GAL1P	Galactose 1-phosphate	2255-14-3
	GL3P	Glycerol 3-phosphate	17989-41-2
	M6P	Mannose 6-phosphate	3672-15-9
	UDP-Glc-Nac	Uridine diphosphate-N-acetylglucosamine	528-04-1
	PRPP	Phosphoribosyl pyrophosphate	7540-64-9
TCA cycle	Cit	Citric acid	77-92-9
	ICit	Isocitric acid	320-77-4
	aKG	a-Ketoglutaric acid	328-50-7
	Suc	Succinic acid	110-15-6
	Fum	Fumaric acid	110-17-8
	Mal	Malic acid	97-67-6
Nucleoside phosphates	AMP	Adenosine monophosphate	61-19-8
	ADP	Adenosine diphosphate	58-64-0
	ATP	Adenosine triphosphate	56-65-5
	GMP	Guanosine monophosphate	85-32-5
	GDP	Guanosine diphosphate	146-91-8
	GTP	Guanosine triphosphate	86-01-1
	CMP	Cytidine monophosphate	63-37-6
	CDP	Cytidine diphosphate	63-38-7
	CTP	Cytidine triphosphate	65-47-4
	UMP	Uridine monophosphate	58-97-9
	UDP	Uridine diphosphate	58-98-0
	UTP	Uridine triphosphate	63-39-8
	IMP	Inosine monophosphate	131-99-7
	ITP	Inosine triphosphate	132-06-9
Cyclic nucleo tides	cAMP	Adenosine 3',5'-cyclic monophosphate	60-92-4
	cGMP	Cyclic guanosine monophosphate	7665-99-8
Deoxynucleotide sugars	dAMP	Deoxyadenosine monophosphate	653-63-4
	dADP	Deoxyadenosine diphosphate	2793-06-8
	dATP	Deoxyadenosine triphosphate	1927-31-7
	dGMP	Deoxyguanosine monophosphate	902-04-5
	dGDP	Deoxyguanosine diphosphate	3493-09-2
	dGTP	Deoxyguanosine triphosphate	2564-35-4
	dCMP	Deoxycytidine monophosphate	1032-65-1
	dCDP	Deoxycytidine diphosphate	800-73-7
	dCTP	Deoxycytidine triphosphate	2056-98-6
	dUMP	Deoxyuridine monophosphate	964-26-1
	dTMP	Deoxythymidine monophosphate	365-07-1
	dTDP	Deoxythymidine diphosphate	491-97-4
	dTTP	Deoxythymidine triphosphate	365-08-2
Amino acids	Ala	Alanine	56-41-7
	Arg	Arginine	74-79-3
	Asn	Asparagine	70-47-3
	Asp	Aspartic acid	56-84-8
	Cys	Cysteine	52-90-4
	Gln	Glutamine	56-85-9
	Glu	Glutamic acid	56-86-0
	Gly	Glycine	56-40-6
	His	Histidine	71-00-1
	Ile	Isoleucine	73-32-5
	Leu	Leucine	61-90-5
	Lys	Lysine	56-87-1
	Met	Methionine	63-68-3
	Phe	Phenylalanine	63-91-2
	Pro	Proline	147-85-3
	Ser	Serine	56-45-1
	Thr	Threonine	72-19-5
	Trp	Tryptophan	73-22-3
	Tyr	Tyrosine	60-18-4
	Val	Valine	72-18-4
