# Supplementary material for: Optimized Fast Filtration-Based Sampling and Extraction Enables Precise and Absolute Quantification of the Escherichia coli Central Carbon Metabolome
Source: Metabolites. 2023 Jan 18;13(2):150. doi: 10.3390/metabo13020150 (PMC9965072; doi:10.3390/metabo13020150)
Supplement: Supplementary file 1 [file metabolites-13-00150-s001.zip › Supplementary Table S2 - Thorfinnsdottir et al.pdf]

Table S2 Intracellular metabolite concentrations in *E. coli* : Intracellular concentrations (mol/L) of all metabolites inculded in the panel for metabolite profiling, listed for all biological replicates. LOQ; limit of quantification. Metabolite abbreviations are listed in Supplementary Table S1.

Media	Cultivation condition	Biological replica	Technical replica	Intracellular concentration (mol/L)											
				2-/3-PG	6PG	ADP	aKG	Ala	AMP	Arg	Asn	Asp	ATP	cAMP	CDP
Mineral	Bioreactor	A	1	3,8E-04	1,0E-04	9,9E-04	8,8E-05	4,7E-04	5,8E-04	2,4E-04	1,5E-04	2,3E-04	3,2E-03	1,3E-05	1,2E-04
			2	2,3E-04	5,8E-05	8,9E-04	1,5E-04	3,3E-04	5,8E-04	1,7E-04	8,6E-05	1,5E-04	2,5E-03	1,5E-05	1,2E-04
			3	2,8E-04	7,1E-05	9,7E-04	1,8E-04	9,8E-04	5,9E-04	4,6E-04	1,8E-04	1,6E-04	2,8E-03	1,2E-05	1,1E-04
			4	2,5E-04	5,1E-05	1,0E-03	1,7E-04	8,0E-04	6,6E-04	3,5E-04	1,4E-04	1,6E-04	2,5E-03	1,1E-05	1,4E-04
		B	1	3,3E-04	9,4E-05	1,0E-03	1,2E-04	3,0E-04	6,2E-04	1,6E-04	1,0E-04	1,6E-04	3,1E-03	1,5E-05	1,3E-04
			2	2,5E-04	7,6E-05	8,2E-04	1,2E-04	2,6E-04	5,2E-04	1,3E-04	7,3E-05	1,2E-04	2,8E-03	1,1E-05	9,7E-05
			3	2,2E-04	5,6E-05	9,3E-04	1,1E-04	3,7E-04	6,0E-04	1,3E-04	8,9E-05	1,6E-04	2,3E-03	1,2E-05	1,3E-04
			4	2,5E-04	5,1E-05	1,0E-03	1,0E-04	3,5E-04	6,8E-04	1,4E-04	8,3E-05	1,6E-04	2,3E-03	9,1E-06	1,6E-04
		C	1	1,9E-04	5,8E-05	5,9E-04	1,4E-04	2,2E-04	3,2E-04	1,1E-04	6,8E-05	9,6E-05	2,5E-03	8,1E-06	7,3E-05
			2	2,4E-04	7,5E-05	6,9E-04	1,1E-04	2,5E-04	4,3E-04	1,4E-04	9,7E-05	1,3E-04	2,4E-03	6,4E-06	8,4E-05
			3	2,2E-04	5,0E-05	7,6E-04	1,1E-04	3,3E-04	5,0E-04	1,4E-04	7,9E-05	1,3E-04	2,2E-03	1,1E-05	1,0E-04
			4	2,2E-04	6,5E-05	7,4E-04	1,1E-04	2,9E-04	4,7E-04	1,3E-04	9,0E-05	1,4E-04	2,5E-03	8,5E-06	9,3E-05
			5	2,6E-04	6,6E-05	7,3E-04	1,3E-04	5,5E-04	4,3E-04	2,3E-04	1,2E-04	1,9E-04	2,7E-03		9,6E-05
			6	2,2E-04	6,2E-05	7,3E-04	1,2E-04	4,1E-04	4,7E-04	1,6E-04	1,2E-04	1,9E-04	2,5E-03		9,8E-05
			7	2,4E-04	6,0E-05	9,1E-04	1,3E-04	3,5E-04	6,7E-04	1,3E-04	9,6E-05	1,6E-04	2,2E-03		1,6E-04
			8	3,1E-04	8,2E-05	9,1E-04	1,2E-04	4,2E-04	5,9E-04	1,5E-04	1,4E-04	2,1E-04	3,0E-03		1,3E-04
			9	3,2E-04	8,5E-05	7,6E-04	9,7E-05	3,7E-04	4,6E-04	1,4E-04	1,3E-04	2,2E-04	2,9E-03		1,0E-04
			10	3,4E-04	1,0E-04	8,6E-04	1,3E-04	4,0E-04	5,5E-04	1,7E-04	1,5E-04	2,3E-04	3,0E-03		1,1E-04
Mineral	Shake flask	A	1	3,3E-04	9,8E-05	1,0E-03	1,2E-04	3,5E-04	7,1E-04	1,6E-04	1,1E-04	2,4E-04	2,9E-03	2,2E-05	1,6E-04
			2	3,8E-04	1,2E-04	1,2E-03	1,6E-04	3,8E-04	6,4E-04	1,5E-04	1,1E-04	1,9E-04	3,5E-03	2,2E-05	1,7E-04
			3	3,5E-04	9,2E-05	1,3E-03	1,6E-04	4,4E-04	9,0E-04	1,6E-04	1,0E-04	2,3E-04	2,9E-03	2,6E-05	1,8E-04
			4	4,4E-04	9,9E-05	1,4E-03	1,4E-04	3,8E-04	1,4E-03	1,5E-04	9,3E-05	2,1E-04	2,4E-03	2,1E-05	2,4E-04
		B	1	3,4E-04	7,2E-05	1,0E-03	1,0E-04	2,9E-04	5,0E-04	1,5E-04	8,7E-05	1,6E-04	3,2E-03	2,2E-05	1,4E-04
			2	2,9E-04	8,4E-05	1,0E-03	1,2E-04	2,8E-04	7,7E-04	1,3E-04	8,4E-05	1,6E-04	2,6E-03	2,6E-05	1,6E-04
			3	2,9E-04	9,2E-05	9,1E-04	1,3E-04	2,7E-04	5,3E-04	1,4E-04	8,3E-05	1,7E-04	3,0E-03	2,1E-05	1,2E-04
			4	3,8E-04	1,3E-04	1,0E-03	1,3E-04	3,4E-04	6,1E-04	1,5E-04	1,2E-04	2,2E-04	3,2E-03	2,6E-05	1,4E-04
		C	1	3,4E-04	1,1E-04	9,4E-04	1,4E-04	3,5E-04	4,9E-04	1,5E-04	1,2E-04	2,2E-04	3,3E-03	2,5E-05	1,3E-04
			2	4,5E-04	1,3E-04	1,3E-03	1,5E-04	4,0E-04	8,1E-04	1,8E-04	1,3E-04	2,8E-04	3,7E-03	2,6E-05	1,9E-04
			3	3,4E-04	1,1E-04	1,0E-03	1,2E-04	3,9E-04	6,0E-04	1,6E-04	1,3E-04	2,4E-04	3,2E-03	1,8E-05	1,4E-04
			4	2,7E-04	7,2E-05	9,5E-04	1,3E-04	3,2E-04	6,1E-04	1,3E-04	9,6E-05	1,8E-04	2,6E-03	1,9E-05	1,3E-04
Rich	Shake flask	A	1	4,1E-04	5,5E-05	1,3E-03	6,2E-05	3,6E-02	1,3E-03	1,8E-02	6,8E-03	3,8E-03	2,0E-03	5,5E-05	9,2E-05
			2	2,6E-04	1,5E-05	1,2E-03	7,7E-05	2,4E-02	1,1E-03	1,2E-02	4,4E-03	2,4E-03	1,4E-03	7,0E-05	6,0E-05
			3	3,8E-04	3,5E-05	1,3E-03	7,7E-05	2,6E-02	1,1E-03	1,4E-02	4,8E-03	2,5E-03	2,3E-03	8,0E-05	7,6E-05
			4	2,7E-04	1,4E-05	1,1E-03	4,5E-05	1,3E-02	1,1E-03	7,1E-03	2,4E-03	1,3E-03	1,5E-03	7,2E-05	5,6E-05

Table S2 Intracellular metabolite concentrations in *E. coli* : Continued

Media	Cultivation condition	Biological replica	Technical replica	Intracellular concentration (mol/L)											
				cGMP	Cit	CMP	CTP	Cys	dADP	dAMP	dATP	dCDP	dCMP	dCTP	dGDP
Mineral	Bioreactor	A	1	2,4E-05	3,5E-04	1,1E-04	2,5E-04	<LOQ	4,7E-05	4,1E-06	1,2E-04	2,5E-05	4,3E-06	9,6E-05	1,1E-05
			2	2,7E-05	3,2E-04	1,0E-04	1,9E-04		4,8E-05	4,9E-06	9,7E-05	2,6E-05	5,1E-06	7,4E-05	1,0E-05
			3	2,4E-05	3,8E-04	1,0E-04	2,3E-04		4,8E-05	4,1E-06	1,1E-04	2,4E-05	4,3E-06	8,9E-05	9,0E-06
			4	2,0E-05	3,6E-04	1,3E-04	1,9E-04		5,8E-05	7,1E-06	9,1E-05	3,2E-05	6,7E-06	7,1E-05	1,3E-05
		B	1	2,7E-05	3,7E-04	1,0E-04	2,3E-04	<LOQ	5,0E-05	4,2E-06	1,2E-04	2,6E-05	4,2E-06	8,6E-05	1,1E-05
			2	2,0E-05	3,2E-04	9,7E-05	2,1E-04		4,0E-05	4,6E-06	1,1E-04	2,0E-05	3,7E-06	7,9E-05	7,2E-06
			3	2,2E-05	2,8E-04	9,2E-05	1,7E-04		5,0E-05	4,8E-06	8,1E-05	2,5E-05	3,9E-06	5,9E-05	9,4E-06
			4	1,5E-05	2,6E-04	1,2E-04	1,6E-04		6,2E-05	5,9E-06	8,4E-05	3,3E-05	6,4E-06	6,1E-05	1,3E-05
		C	1	1,9E-05	2,7E-04	8,2E-05	2,0E-04	<LOQ	2,8E-05	2,7E-06	9,0E-05	1,6E-05	3,3E-06	6,6E-05	9,8E-06
			2	1,8E-05	3,1E-04	9,5E-05	2,1E-04		3,3E-05	3,5E-06	9,7E-05	1,7E-05	3,8E-06	8,2E-05	1,1E-05
			3	2,5E-05	2,8E-04	8,8E-05	2,0E-04		3,6E-05	4,5E-06	8,1E-05	1,8E-05	3,8E-06	6,4E-05	1,0E-05
			4	2,1E-05	2,9E-04	9,2E-05	2,2E-04		3,4E-05	2,9E-06	9,2E-05	1,6E-05	3,3E-06	7,3E-05	8,6E-06
			5		3,7E-04	9,3E-05	2,5E-04		3,3E-05	2,9E-06	9,9E-05	1,7E-05	3,5E-06	8,3E-05	8,2E-06
			6		2,8E-04	9,4E-05	2,3E-04		3,0E-05	3,2E-06	9,1E-05	1,6E-05	3,5E-06	7,6E-05	7,6E-06
			7		2,6E-04	1,3E-04	1,9E-04		5,2E-05	4,5E-06	8,1E-05	2,9E-05	4,8E-06	6,5E-05	1,2E-05
			8		3,7E-04	1,1E-04	2,8E-04		3,9E-05	3,5E-06	1,1E-04	2,1E-05	3,9E-06	8,7E-05	8,6E-06
			9		3,3E-04	9,4E-05	2,5E-04		3,4E-05	2,6E-06	9,9E-05	1,8E-05	3,2E-06	8,3E-05	9,1E-06
			10		3,4E-04	1,0E-04	2,7E-04		3,8E-05	3,0E-06	1,1E-04	1,8E-05	2,7E-06	9,1E-05	8,8E-06
Mineral	Shake flask	A	1	3,6E-05	1,3E-04	1,4E-04	2,2E-04	<LOQ	5,2E-05	7,0E-06	9,9E-05	3,0E-05	6,8E-06	7,6E-05	1,4E-05
			2	3,5E-05	1,8E-04	1,3E-04	2,6E-04		5,6E-05	6,0E-06	1,2E-04	3,0E-05	5,4E-06	8,3E-05	1,2E-05
			3	4,5E-05	1,7E-04	1,8E-04	2,2E-04		6,7E-05	7,9E-06	1,0E-04	3,4E-05	9,4E-06	7,1E-05	1,6E-05
			4	3,6E-05	1,6E-04	2,3E-04	1,8E-04		8,2E-05	1,3E-05	8,9E-05	4,7E-05	1,3E-05	6,6E-05	2,5E-05
		B	1	3,9E-05	2,7E-04	1,2E-04	2,3E-04	<LOQ	4,2E-05	5,1E-06	1,1E-04	2,6E-05	4,7E-06	7,8E-05	1,2E-05
			2	5,0E-05	1,5E-04	1,5E-04	1,8E-04		5,8E-05	8,9E-06	9,2E-05	3,1E-05	7,8E-06	6,6E-05	1,2E-05
			3	3,7E-05	1,5E-04	1,2E-04	2,3E-04		4,2E-05	4,9E-06	1,1E-04	2,3E-05	5,5E-06	7,6E-05	1,0E-05
			4	4,7E-05	1,4E-04	1,2E-04	2,5E-04		4,7E-05	5,5E-06	1,1E-04	2,6E-05	5,3E-06	8,8E-05	1,1E-05
		C	1	4,9E-05	1,5E-04	1,2E-04	2,5E-04	<LOQ	4,5E-05	6,8E-06	1,2E-04	2,5E-05	6,3E-06	8,0E-05	1,8E-05
			2	4,7E-05	1,9E-04	1,8E-04	2,8E-04		6,6E-05	1,1E-05	1,3E-04	3,5E-05	8,9E-06	9,2E-05	2,4E-05
			3	2,9E-05	1,6E-04	1,2E-04	2,5E-04		4,6E-05	6,5E-06	1,2E-04	2,3E-05	6,1E-06	7,3E-05	1,6E-05
			4	3,1E-05	1,4E-04	1,2E-04	1,9E-04		4,6E-05	7,2E-06	9,4E-05	2,3E-05	6,3E-06	5,8E-05	1,4E-05
Rich	Shake flask	A	1	7,4E-05	4,4E-04	8,8E-05	7,5E-05	<LOQ	6,9E-05	9,5E-06	8,3E-05	6,0E-05	1,4E-05	9,1E-05	2,6E-05
			2	8,5E-05	2,8E-04	7,5E-05	3,5E-05		7,6E-05	1,5E-05	6,5E-05	5,4E-05	2,1E-05	5,2E-05	2,9E-05
			3	9,7E-05	3,3E-04	7,1E-05	7,6E-05		6,8E-05	1,2E-05	9,4E-05	4,9E-05	1,4E-05	9,5E-05	2,7E-05
			4	8,7E-05	2,1E-04	6,7E-05	3,8E-05		7,2E-05	2,1E-05	6,7E-05	4,6E-05	2,0E-05	5,6E-05	3,0E-05

Table S2 Intracellular metabolite concentrations in *E. coli* : Continued

Media	Cultivation condition	Biological replica	Technical replica	Intracellular concentration (mol/L)											
				**dGMP**	**dGTP**	**dTDP**	**dTMP**	**dTTP**	**dUMP**	**F1,6BP**	**F1P**	**F6P**	**FAD**	**Fum**	**G-/M-1P**
Mineral	Bioreactor	A	1	<LOQ	4,1E-05	4,6E-05	1,3E-03	1,3E-04	2,9E-06	2,5E-03	3,8E-05	4,4E-04	9,2E-05	2,2E-04	3,2E-05
			2		3,1E-05	4,6E-05	1,0E-03	1,0E-04	1,8E-06	1,0E-03	2,5E-05	3,9E-04	8,7E-05	2,2E-04	2,5E-05
			3		3,3E-05	4,6E-05	1,2E-03	1,2E-04	2,4E-06	1,2E-03	2,5E-05	3,1E-04	7,9E-05	2,3E-04	3,2E-05
			4		2,8E-05	5,6E-05	1,1E-03	1,0E-04	2,6E-06	7,9E-04	2,4E-05	3,3E-04	8,6E-05	2,5E-04	2,8E-05
		B	1	<LOQ	3,9E-05	4,6E-05	1,3E-03	1,2E-04	3,3E-06	1,4E-03	3,5E-05	3,9E-04	8,0E-05	2,9E-04	3,7E-05
			2		3,3E-05	3,7E-05	1,2E-03	1,1E-04	2,0E-06	1,0E-03	2,6E-05	3,1E-04	8,6E-05	2,5E-04	3,1E-05
			3		2,5E-05	4,4E-05	8,3E-04	8,2E-05	1,3E-06	6,9E-04	2,0E-05	4,8E-04	8,5E-05	2,5E-04	2,7E-05
			4		2,7E-05	5,5E-05	8,8E-04	8,6E-05	1,5E-06	7,3E-04	2,2E-05	4,8E-04	8,5E-05	2,4E-04	2,7E-05
		C	1	<LOQ	3,0E-05	2,8E-05	1,1E-03	1,0E-04	3,0E-06	6,6E-04	2,2E-05	2,6E-04	5,9E-05	2,5E-04	3,2E-05
			2		3,6E-05	3,0E-05	1,2E-03	1,1E-04	2,4E-06	1,5E-03	2,9E-05	3,0E-04	6,1E-05	2,6E-04	2,7E-05
			3		2,8E-05	3,7E-05	1,0E-03	9,4E-05	2,6E-06	6,6E-04	2,0E-05	3,3E-04	5,9E-05	2,3E-04	2,9E-05
			4		3,0E-05	3,4E-05	1,2E-03	1,1E-04	3,0E-06	8,1E-04	2,4E-05	2,9E-04	6,4E-05	2,4E-04	3,0E-05
			5		3,3E-05	3,5E-05	1,2E-03	1,2E-04	3,5E-06	1,0E-03	2,7E-05	3,4E-04		2,6E-04	3,4E-05
			6		2,9E-05	3,6E-05	1,2E-03	1,1E-04	3,0E-06	8,0E-04	2,2E-05	2,7E-04		2,2E-04	3,0E-05
			7		2,4E-05	5,9E-05	1,2E-03	9,9E-05	3,5E-06	7,9E-04	2,1E-05	2,6E-04		2,4E-04	2,9E-05
			8		3,3E-05	4,5E-05	1,4E-03	1,3E-04	3,6E-06	1,2E-03	3,1E-05	3,5E-04		2,6E-04	3,8E-05
			9		3,4E-05	3,9E-05	1,2E-03	1,3E-04	3,3E-06	1,6E-03	3,5E-05	2,9E-04		2,4E-04	3,4E-05
			10		3,6E-05	3,9E-05	1,3E-03	1,4E-04	3,9E-06	1,9E-03	3,7E-05	3,2E-04		2,6E-04	3,5E-05
Mineral	Shake flask	A	1	<LOQ	4,0E-05	5,1E-05	1,1E-03	1,1E-04	2,5E-06	1,3E-03	3,1E-05	5,8E-04	8,5E-05	2,5E-04	2,7E-05
			2		4,2E-05	5,6E-05	1,3E-03	1,3E-04	2,7E-06	1,3E-03	3,0E-05	4,9E-04	9,0E-05	2,5E-04	3,7E-05
			3		3,4E-05	6,7E-05	1,2E-03	1,1E-04	2,1E-06	9,0E-04	2,5E-05	9,9E-04	8,8E-05	2,6E-04	2,8E-05
			4		3,2E-05	9,7E-05	1,3E-03	1,1E-04	2,7E-06	1,3E-03	2,9E-05	7,1E-04	8,6E-05	2,7E-04	2,8E-05
		B	1	<LOQ	3,9E-05	4,6E-05	1,2E-03	1,2E-04	3,0E-06	1,0E-03	2,3E-05	4,2E-04	7,7E-05	2,5E-04	3,1E-05
			2		3,1E-05	5,9E-05	1,2E-03	1,0E-04	2,8E-06	8,3E-04	2,2E-05	6,9E-04	7,9E-05	2,4E-04	2,6E-05
			3		3,7E-05	4,5E-05	1,2E-03	1,2E-04	2,8E-06	1,1E-03	2,4E-05	3,3E-04	8,6E-05	2,3E-04	3,1E-05
			4		4,1E-05	4,9E-05	1,3E-03	1,3E-04	2,8E-06	1,7E-03	2,9E-05	3,4E-04	8,4E-05	2,4E-04	3,7E-05
		C	1	<LOQ	4,6E-05	4,1E-05	1,3E-03	1,2E-04	4,4E-06	1,1E-03	3,6E-05	4,2E-04	8,0E-05	3,9E-04	3,8E-05
			2		5,1E-05	6,2E-05	1,6E-03	1,4E-04	4,4E-06	1,4E-03	4,3E-05	6,7E-04	7,8E-05	3,9E-04	4,1E-05
			3		4,4E-05	4,3E-05	1,2E-03	1,2E-04	3,0E-06	1,2E-03	3,3E-05	4,5E-04	8,4E-05	3,3E-04	3,9E-05
			4		3,2E-05	4,6E-05	1,0E-03	1,0E-04	2,7E-06	7,7E-04	2,4E-05	4,4E-04	7,7E-05	3,0E-04	2,8E-05
Rich	Shake flask	A	1	<LOQ	4,4E-05	6,8E-05	8,8E-04	1,1E-04	3,3E-06	5,7E-05	1,6E-05	1,6E-04	6,1E-05	8,9E-04	2,1E-05
			2		3,6E-05	7,1E-05	7,7E-04	7,9E-05	1,6E-06	3,8E-05	9,0E-06	4,7E-05	6,0E-05	6,2E-04	8,6E-06
			3		5,1E-05	6,4E-05	9,0E-04	1,2E-04	2,6E-06	6,4E-05	1,1E-05	8,7E-05	6,5E-05	6,2E-04	1,6E-05
			4		4,2E-05	6,2E-05	8,5E-04	8,6E-05	1,2E-06	3,6E-05	7,7E-06	3,1E-05	6,4E-05	3,5E-04	7,9E-06

Table S2 Intracellular metabolite concentrations in *E. coli* : Continued

Media	Cultivation condition	Biological replica	Technical replica	Intracellular concentration (mol/L)											
				**G6P**	**GA6P**	**GAL1P**	**GDP**	**GL3P**	**Gln**	**Glu**	**Gly**	**GMP**	**GTP**	**His**	**ICit**
Mineral	Bioreactor	A	1	4,8E-04	2,0E-04	1,0E-05	1,7E-04	1,2E-04	7,1E-04	2,4E-03	5,0E-04	2,1E-05	1,1E-03	7,9E-05	3,9E-06
			2	4,5E-04	1,3E-04	6,5E-06	1,8E-04	1,3E-04	3,2E-04	1,6E-03	3,9E-04	2,8E-05	9,1E-04	6,3E-05	4,3E-06
			3	3,6E-04	1,3E-04	1,1E-05	1,8E-04	8,3E-05	3,6E-04	2,1E-03	7,8E-04	2,2E-05	1,1E-03	1,7E-04	5,7E-06
			4	4,6E-04	1,3E-04	9,4E-06	2,2E-04	2,0E-04	3,0E-04	2,1E-03	6,3E-04	4,6E-05	9,2E-04	1,3E-04	3,9E-06
		B	1	4,4E-04	1,9E-04	1,0E-05	2,1E-04	1,2E-04	3,7E-04	2,0E-03	4,1E-04	2,4E-05	1,1E-03	8,7E-05	3,9E-06
			2	3,3E-04	1,3E-04	6,8E-06	1,7E-04	9,2E-05	2,2E-04	1,5E-03	3,8E-04	2,1E-05	1,0E-03	6,4E-05	3,7E-06
			3	5,6E-04	1,3E-04	4,8E-06	2,0E-04	2,0E-04	3,8E-04	1,9E-03	4,1E-04	3,1E-05	8,2E-04	6,7E-05	2,2E-06
			4	5,8E-04	1,3E-04	4,6E-06	2,4E-04	2,0E-04	3,3E-04	1,9E-03	4,4E-04	4,6E-05	8,3E-04	7,1E-05	1,5E-06
		C	1	2,9E-04	1,1E-04	7,4E-06	1,8E-04	9,9E-05	2,3E-04	1,3E-03	3,0E-04	1,6E-05	8,3E-04	4,8E-05	4,1E-06
			2	2,9E-04	1,5E-04	8,5E-06	2,0E-04	1,1E-04	3,0E-04	1,2E-03	2,7E-04	1,4E-05	8,6E-04	5,0E-05	4,8E-06
			3	4,4E-04	1,3E-04	6,8E-06	2,0E-04	1,3E-04	3,0E-04	1,7E-03	3,6E-04	2,0E-05	7,9E-04	6,3E-05	2,9E-06
			4	3,2E-04	1,3E-04	7,7E-06	1,8E-04	1,2E-04	3,3E-04	1,8E-03	3,5E-04	1,5E-05	8,7E-04	6,0E-05	3,5E-06
			5	3,4E-04	1,3E-04	9,5E-06	1,7E-04	1,3E-04	3,7E-04	2,2E-03	5,3E-04	1,4E-05	9,6E-04	9,1E-05	5,4E-06
			6	2,9E-04	1,1E-04	7,3E-06	1,6E-04	1,1E-04	4,4E-04	2,3E-03	5,0E-04	1,5E-05	8,6E-04	8,2E-05	3,9E-06
			7	2,9E-04	1,3E-04	8,6E-06	2,4E-04	1,2E-04	3,7E-04	2,1E-03	4,2E-04	2,9E-05	7,4E-04	6,9E-05	3,2E-06
			8	4,1E-04	1,9E-04	1,1E-05	1,9E-04	1,5E-04	5,8E-04	2,6E-03	4,4E-04	1,7E-05	9,8E-04	7,2E-05	3,8E-06
			9	3,4E-04	2,0E-04	8,3E-06	1,6E-04	1,3E-04	5,5E-04	2,3E-03	4,5E-04	1,3E-05	9,3E-04	8,3E-05	3,6E-06
			10	3,9E-04	2,1E-04	9,4E-06	1,7E-04	1,3E-04	5,6E-04	2,6E-03	4,3E-04	1,3E-05	1,0E-03	6,8E-05	4,4E-06
Mineral	Shake flask	A	1	7,2E-04	2,0E-04	9,7E-06	2,3E-04	2,0E-04	6,4E-04	2,7E-03	4,0E-04	4,6E-05	1,0E-03	6,5E-05	1,7E-06
			2	5,7E-04	2,2E-04	1,2E-05	2,3E-04	1,4E-04	5,9E-04	2,7E-03	4,1E-04	3,4E-05	1,2E-03	6,8E-05	2,5E-06
			3	1,2E-03	1,9E-04	1,1E-05	2,7E-04	2,4E-04	5,9E-04	2,8E-03	4,7E-04	7,2E-05	1,0E-03	7,8E-05	2,1E-06
			4	9,7E-04	1,9E-04	9,3E-06	3,9E-04	3,3E-04	5,8E-04	2,5E-03	4,2E-04	1,2E-04	9,4E-04	6,8E-05	1,7E-06
		B	1	5,3E-04	2,0E-04	9,5E-06	2,1E-04	1,6E-04	4,3E-04	2,0E-03	3,6E-04	3,4E-05	1,1E-03	5,7E-05	2,7E-06
			2	8,9E-04	1,9E-04	8,2E-06	2,3E-04	2,2E-04	4,6E-04	2,0E-03	3,3E-04	5,7E-05	8,8E-04	5,4E-05	1,7E-06
			3	4,0E-04	1,9E-04	9,1E-06	1,8E-04	1,2E-04	4,2E-04	2,0E-03	3,5E-04	2,4E-05	1,0E-03	6,0E-05	3,0E-06
			4	4,2E-04	1,9E-04	1,1E-05	2,1E-04	1,3E-04	6,7E-04	2,6E-03	3,7E-04	2,7E-05	1,1E-03	6,5E-05	2,7E-06
		C	1	4,8E-04	2,2E-04	1,2E-05	3,4E-04	1,8E-04	5,7E-04	2,5E-03	3,8E-04	2,9E-05	1,1E-03	6,9E-05	3,4E-06
			2	8,4E-04	2,7E-04	1,3E-05	4,4E-04	2,8E-04	7,4E-04	3,0E-03	4,7E-04	5,4E-05	1,3E-03	7,7E-05	2,8E-06
			3	5,3E-04	2,2E-04	9,1E-06	3,0E-04	1,8E-04	7,6E-04	2,9E-03	3,9E-04	2,7E-05	1,1E-03	7,4E-05	3,0E-06
			4	4,9E-04	1,6E-04	8,2E-06	2,8E-04	1,8E-04	4,9E-04	2,2E-03	3,6E-04	3,5E-05	9,2E-04	6,0E-05	1,9E-06
Rich	Shake flask	A	1	1,7E-04	1,9E-04	3,8E-05	1,8E-04	1,6E-04	4,2E-04	2,7E-02	1,6E-02	8,0E-05	5,4E-04	3,4E-03	8,4E-06
			2	5,9E-05	9,9E-05	2,1E-05	1,7E-04	1,2E-04	1,0E-04	1,8E-02	1,1E-02	1,1E-04	4,0E-04	2,2E-03	4,2E-06
			3	9,2E-05	1,5E-04	2,5E-05	1,8E-04	1,1E-04	3,2E-04	1,9E-02	1,2E-02	7,2E-05	6,5E-04	2,5E-03	6,5E-06
			4	5,1E-05	9,7E-05	1,6E-05	1,8E-04	1,0E-04	9,1E-05	9,5E-03	6,3E-03	8,0E-05	4,6E-04	1,3E-03	4,2E-06

Table S2 Intracellular metabolite concentrations in *E. coli* : Continued

Media	Cultivation condition	Biological replica	Technical replica	Intracellular concentration (mol/L)											
				**Ile**	**IMP**	**ITP**	**Leu**	**Lys**	**M6P**	**Mal**	**Met**	**NAD**	**NADH**	**NADP**	**NADPH**
Mineral	Bioreactor	A	1	5,9E-05	1,7E-04	<LOQ	1,8E-04	4,6E-04	1,1E-04	3,7E-04	8,7E-05	1,1E-03	2,8E-04	1,5E-04	8,9E-05
			2	4,0E-05	1,7E-04		1,5E-04	3,9E-04	9,4E-05	3,2E-04	8,1E-05	9,7E-04	2,3E-04	1,5E-04	8,1E-05
			3	2,6E-04	1,7E-04		5,0E-04	1,4E-03	1,2E-04	4,1E-04	1,8E-04	9,5E-04	2,3E-04	1,4E-04	8,7E-05
			4	1,9E-04	2,1E-04		3,9E-04	9,5E-04	1,1E-04	4,0E-04	1,4E-04	9,9E-04	2,2E-04	1,8E-04	9,0E-05
		B	1	3,0E-05	1,9E-04	<LOQ	1,2E-04	4,0E-04	1,3E-04	3,9E-04	7,2E-05	1,0E-03	2,2E-04	1,8E-04	7,4E-05
			2	3,5E-05	1,6E-04		1,2E-04	3,5E-04	1,1E-04	2,9E-04	6,6E-05	9,5E-04	2,1E-04	1,8E-04	6,7E-05
			3	2,5E-05	1,9E-04		1,2E-04	3,6E-04	1,0E-04	3,0E-04	6,5E-05	9,0E-04	2,1E-04	1,9E-04	7,3E-05
			4	2,7E-05	2,1E-04		1,2E-04	3,7E-04	1,1E-04	2,9E-04	6,8E-05	8,7E-04	1,9E-04	1,7E-04	7,2E-05
		C	1	2,4E-05	9,1E-05	<LOQ	9,7E-05	2,9E-04	9,9E-05	2,5E-04	6,0E-05	7,5E-04	1,7E-04	1,0E-04	5,7E-05
			2	2,2E-05	1,1E-04		7,3E-05	3,1E-04	9,3E-05	3,2E-04	6,5E-05	4,4E-04	1,1E-04	7,6E-05	4,4E-05
			3	3,6E-05	1,3E-04		1,3E-04	3,7E-04	9,9E-05	2,8E-04	6,9E-05	6,9E-04	1,5E-04	1,0E-04	5,6E-05
			4	2,7E-05	1,3E-04		1,2E-04	3,3E-04	1,0E-04	2,9E-04	6,2E-05	6,7E-04	1,4E-04	1,1E-04	5,1E-05
			5	9,2E-05	1,3E-04		2,5E-04	5,1E-04	1,0E-04	3,9E-04	8,5E-05				
			6	3,4E-05	1,3E-04		1,6E-04	4,3E-04	1,0E-04	2,7E-04	8,3E-05				
			7	2,9E-05	1,9E-04		1,4E-04	3,4E-04	9,0E-05	3,0E-04	6,6E-05				
			8	2,6E-05	1,6E-04		1,4E-04	3,8E-04	1,2E-04	4,1E-04	7,2E-05				
			9	2,6E-05	1,4E-04		1,2E-04	3,4E-04	9,8E-05	3,6E-04	6,7E-05				
			10	2,8E-05	1,5E-04		1,2E-04	3,8E-04	1,1E-04	4,6E-04	7,1E-05				
Mineral	Shake flask	A	1	2,3E-05	2,2E-04	<LOQ	8,1E-05	3,1E-04	1,5E-04	3,6E-04	6,5E-05	5,7E-04	4,4E-04	2,1E-04	8,1E-05
			2	2,1E-05	2,3E-04		8,7E-05	3,4E-04	1,6E-04	3,8E-04	7,1E-05	7,0E-04	5,2E-04	2,2E-04	8,5E-05
			3	3,1E-05	2,8E-04		1,1E-04	3,6E-04	1,9E-04	4,1E-04	7,1E-05	6,6E-04	5,1E-04	2,4E-04	8,7E-05
			4	2,4E-05	3,2E-04		9,0E-05	3,5E-04	1,8E-04	4,0E-04	6,7E-05	6,7E-04	4,8E-04	2,5E-04	8,0E-05
		B	1	1,9E-05	2,2E-04	<LOQ	8,7E-05	3,1E-04	1,2E-04	2,8E-04	6,4E-05	5,5E-04	3,6E-04	1,8E-04	5,9E-05
			2	1,5E-05	2,3E-04		7,3E-05	3,0E-04	1,4E-04	3,0E-04	5,8E-05	5,5E-04	4,0E-04	1,6E-04	9,2E-05
			3	2,1E-05	1,8E-04		9,6E-05	3,0E-04	1,2E-04	3,1E-04	6,5E-05	5,0E-04	3,4E-04	2,0E-04	6,6E-05
			4	1,9E-05	1,9E-04		7,5E-05	3,1E-04	1,4E-04	3,6E-04	6,8E-05	6,0E-04	4,2E-04	2,1E-04	7,4E-05
		C	1	2,4E-05	1,6E-04	<LOQ	8,8E-05	3,3E-04	1,6E-04	3,7E-04	6,8E-05	6,2E-04	4,0E-04	2,0E-04	7,0E-05
			2	2,3E-05	2,2E-04		9,1E-05	3,9E-04	2,1E-04	4,5E-04	8,0E-05	5,2E-04	4,2E-04	1,7E-04	8,6E-05
			3	1,6E-05	2,0E-04		7,0E-05	3,3E-04	1,5E-04	3,9E-04	6,8E-05	5,5E-04	3,6E-04	2,1E-04	5,9E-05
			4	1,7E-05	2,3E-04		7,3E-05	3,0E-04	1,4E-04	3,4E-04	6,2E-05	4,9E-04	3,3E-04	2,0E-04	5,4E-05
Rich	Shake flask	A	1	1,2E-02	2,8E-04	<LOQ	2,0E-02	1,5E-02	8,0E-05	9,7E-04	3,8E-03	1,4E-03	2,1E-04	1,3E-04	6,2E-05
			2	7,9E-03	2,8E-04		1,4E-02	1,1E-02	2,6E-05	6,5E-04	2,5E-03	1,3E-03	1,8E-04	1,2E-04	5,5E-05
			3	8,4E-03	3,0E-04		1,4E-02	1,2E-02	4,6E-05	7,9E-04	2,7E-03	1,4E-03	1,9E-04	1,4E-04	5,2E-05
			4	4,3E-03	2,7E-04		7,5E-03	5,9E-03	2,4E-05	4,7E-04	1,3E-03	1,4E-03	2,0E-04	1,4E-04	6,3E-05

Table S2 Intracellular metabolite concentrations in *E. coli* : Continued

Media	Cultivation condition	Biological replica	Technical replica	Intracellular concentration (mol/L)											
				**PEP**	**Phe**	**Pro**	**PRPP**	**R5P**	**S7P**	**Ser**	**Suc**	**Thr**	**Trp**	**Tyr**	**UDP**
Mineral	Bioreactor	A	1	8,3E-05	1,2E-04	1,6E-04	1,2E-04	2,2E-04	7,0E-05	2,8E-04	1,8E-03	2,6E-04	3,7E-05	1,7E-04	2,6E-04
			2	7,1E-05	9,2E-05	1,2E-04	5,0E-05	1,7E-04	5,6E-05	2,5E-04	1,4E-03	1,9E-04	2,5E-05	1,2E-04	2,7E-04
			3	1,1E-04	4,3E-04	2,0E-04	8,0E-05	1,5E-04	6,1E-05	2,6E-04	2,0E-03	3,7E-04	6,5E-05	1,5E-04	2,8E-04
			4	8,8E-05	2,6E-04	1,6E-04	5,5E-05	1,7E-04	5,4E-05	2,4E-04	1,5E-03	2,9E-04	4,7E-05	1,3E-04	3,6E-04
		B	1	1,1E-04	7,9E-05	1,2E-04	1,1E-04	2,2E-04	8,2E-05	2,6E-04	2,2E-03	2,2E-04	2,8E-05	1,3E-04	2,8E-04
			2	8,2E-05	7,7E-05	1,0E-04	7,9E-05	1,8E-04	7,1E-05	2,7E-04	2,1E-03	1,6E-04	2,4E-05	9,9E-05	2,2E-04
			3	5,5E-05	9,3E-05	1,2E-04	6,0E-05	1,7E-04	6,0E-05	2,5E-04	1,6E-03	2,0E-04	2,9E-05	1,3E-04	3,0E-04
			4	5,3E-05	8,9E-05	1,2E-04	5,2E-05	1,9E-04	6,3E-05	3,0E-04	1,7E-03	2,2E-04	2,6E-05	1,3E-04	3,4E-04
		C	1	1,5E-04	6,8E-05	8,6E-05	9,1E-05	1,1E-04	5,7E-05	1,7E-04	1,7E-03	1,4E-04	2,1E-05	9,4E-05	1,8E-04
			2	1,4E-04	5,5E-05	9,0E-05	4,4E-05	1,5E-04	5,5E-05	1,8E-04	1,9E-03	1,7E-04	2,2E-05	9,4E-05	1,8E-04
			3	1,3E-04	8,8E-05	9,8E-05	7,6E-05	1,4E-04	5,7E-05	2,2E-04	1,4E-03	1,9E-04	2,5E-05	1,1E-04	2,4E-04
			4	1,2E-04	8,0E-05	9,6E-05	1,0E-04	1,5E-04	6,0E-05	2,3E-04	1,7E-03	1,9E-04	2,6E-05	1,2E-04	2,2E-04
			5	1,4E-04	1,4E-04	1,4E-04	7,8E-05	1,4E-04	6,1E-05	3,3E-04	1,9E-03	2,6E-04	3,5E-05	1,5E-04	2,3E-04
			6	1,2E-04	1,2E-04	1,4E-04	8,6E-05	1,4E-04	6,0E-05	3,4E-04	1,7E-03	2,6E-04	3,3E-05	1,6E-04	2,3E-04
			7	1,2E-04	1,0E-04	1,1E-04	8,2E-05	1,6E-04	6,3E-05	3,1E-04	1,6E-03	2,1E-04	3,2E-05	1,4E-04	4,0E-04
			8	1,4E-04	1,1E-04	1,3E-04	1,4E-04	1,9E-04	7,0E-05	2,7E-04	2,1E-03	2,5E-04	3,6E-05	1,6E-04	2,9E-04
			9	1,3E-04	8,8E-05	1,3E-04	1,4E-04	1,8E-04	6,5E-05	3,4E-04	1,7E-03	2,5E-04	3,3E-05	1,4E-04	2,3E-04
			10	1,3E-04	9,4E-05	1,4E-04	1,4E-04	2,1E-04	7,2E-05	2,5E-04	2,0E-03	2,5E-04	3,1E-05	1,6E-04	2,4E-04
Mineral	Shake flask	A	1	8,0E-05	5,5E-05	8,8E-05	1,2E-04	2,2E-04	6,9E-05	2,7E-04	1,6E-03	2,4E-04	2,2E-05	1,5E-04	3,2E-04
			2	1,1E-04	6,3E-05	9,7E-05	8,4E-05	2,3E-04	8,6E-05	2,9E-04	1,8E-03	2,5E-04	2,3E-05	1,6E-04	3,6E-04
			3	1,1E-04	7,4E-05	1,0E-04	6,9E-05	2,1E-04	7,9E-05	3,4E-04	1,7E-03	2,5E-04	2,6E-05	1,6E-04	4,2E-04
			4	1,4E-04	5,7E-05	9,5E-05	7,8E-05	2,7E-04	8,3E-05	3,2E-04	1,9E-03	2,3E-04	2,3E-05	1,6E-04	6,1E-04
		B	1	1,3E-04	5,2E-05	9,3E-05	6,0E-05	2,0E-04	6,7E-05	2,7E-04	1,5E-03	1,9E-04	1,9E-05	1,4E-04	3,0E-04
			2	9,8E-05	4,9E-05	7,9E-05	3,9E-05	2,1E-04	6,6E-05	2,3E-04	1,4E-03	1,9E-04	1,8E-05	1,4E-04	3,9E-04
			3	8,4E-05	5,4E-05	9,6E-05	4,9E-05	2,1E-04	6,9E-05	2,7E-04	1,7E-03	1,8E-04	1,9E-05	1,3E-04	2,8E-04
			4	8,3E-05	5,4E-05	8,3E-05	1,1E-04	2,4E-04	7,7E-05	2,7E-04	1,9E-03	2,2E-04	2,3E-05	1,5E-04	3,1E-04
		C	1	2,3E-04	5,9E-05	1,0E-04	6,4E-05	1,8E-04	8,0E-05	2,5E-04	2,0E-03	2,2E-04	2,3E-05	1,6E-04	3,1E-04
			2	2,6E-04	6,9E-05	1,1E-04	1,5E-04	2,4E-04	1,0E-04	3,6E-04	2,7E-03	2,7E-04	2,9E-05	1,9E-04	4,2E-04
			3	1,7E-04	5,3E-05	8,5E-05	9,3E-05	2,3E-04	8,0E-05	2,5E-04	2,2E-03	2,3E-04	2,5E-05	1,7E-04	2,8E-04
			4	1,3E-04	5,5E-05	7,6E-05	6,1E-05	1,8E-04	7,0E-05	2,5E-04	1,7E-03	2,0E-04	2,0E-05	1,5E-04	2,9E-04
Rich	Shake flask	A	1	3,6E-04	1,1E-02	5,5E-03	1,4E-05	7,1E-05	5,9E-05	2,3E-04	2,8E-03	8,7E-03	2,1E-03	2,3E-03	3,3E-04
			2	2,5E-04	7,4E-03	3,8E-03	2,1E-06	5,0E-05	3,3E-05	1,9E-04	2,0E-03	5,2E-03	1,3E-03	1,6E-03	3,8E-04
			3	3,8E-04	8,2E-03	4,1E-03	4,9E-06	5,3E-05	4,4E-05	1,8E-04	2,6E-03	5,7E-03	1,3E-03	1,7E-03	3,3E-04
			4	2,7E-04	4,1E-03	2,0E-03	1,6E-06	4,0E-05	3,3E-05	1,2E-04	2,0E-03	2,9E-03	5,9E-04	8,2E-04	3,5E-04

Table S2 Intracellular metabolite concentrations in *E. coli* : Continued

Media	Cultivation condition	Biological replica	Technical replica	Intracellular concentration (mol/L)			
				UDP-GlcNac	UMP	UTP	Val
Mineral	Bioreactor	A	1	7,6E-04	3,2E-03	1,2E-03	2,3E-04
			2	6,7E-04	2,5E-03	9,5E-04	1,6E-04
			3	7,9E-04	2,9E-03	1,2E-03	4,6E-04
			4	7,2E-04	2,5E-03	1,0E-03	3,6E-04
		B	1	7,9E-04	3,1E-03	1,2E-03	1,6E-04
			2	7,7E-04	2,8E-03	1,1E-03	1,4E-04
			3	5,3E-04	1,9E-03	9,0E-04	1,7E-04
			4	5,7E-04	2,2E-03	8,2E-04	1,5E-04
		C	1	8,1E-04	2,3E-03	9,9E-04	1,3E-04
			2	8,6E-04	2,6E-03	9,8E-04	1,1E-04
			3	7,3E-04	2,2E-03	9,4E-04	1,6E-04
			4	8,7E-04	2,5E-03	1,1E-03	1,5E-04
			5	9,1E-04	2,6E-03	1,3E-03	2,7E-04
			6	8,8E-04	2,5E-03	1,2E-03	2,0E-04
			7	8,3E-04	2,4E-03	1,0E-03	1,8E-04
			8	9,4E-04	2,9E-03	1,4E-03	2,0E-04
			9	8,2E-04	2,9E-03	1,2E-03	1,8E-04
			10	8,7E-04	3,1E-03	1,3E-03	1,6E-04
Mineral	Shake flask	A	1	7,0E-04	2,9E-03	1,0E-03	1,6E-04
			2	8,0E-04	3,3E-03	1,3E-03	1,7E-04
			3	6,9E-04	2,9E-03	1,1E-03	1,8E-04
			4	8,1E-04	3,1E-03	9,3E-04	1,6E-04
		B	1	7,6E-04	2,9E-03	1,2E-03	1,6E-04
			2	7,1E-04	2,8E-03	9,9E-04	1,3E-04
			3	7,5E-04	2,9E-03	1,2E-03	1,4E-04
			4	7,7E-04	3,1E-03	1,2E-03	1,5E-04
		C	1	9,9E-04	2,9E-03	1,2E-03	1,6E-04
			2	1,1E-03	3,6E-03	1,3E-03	1,9E-04
			3	9,2E-04	2,9E-03	1,1E-03	1,6E-04
			4	7,4E-04	2,4E-03	9,3E-04	1,3E-04
Rich	Shake flask	A	1	7,7E-04	2,1E-03	6,7E-04	1,5E-02
			2	7,8E-04	1,6E-03	5,4E-04	1,0E-02
			3	9,0E-04	2,0E-03	7,5E-04	1,1E-02
			4	8,3E-04	1,7E-03	5,5E-04	5,3E-03
